# RETRACTION NOTE TO: Comparing sequential *vs.* day 3 *vs.* day 5 embryo transfers in cases with recurrent implantation failure: randomized controlled trial

**DOI:** 10.5935/1518-0557.20200083ra

**Published:** 2023

**Authors:** Haitham Torky, Ali Ahmad, Ahmed Hussein, El-Sayed El-Desouky, Rania Aly, Mona Ragab, Ashraf Abo-Louz

**Affiliations:** 1 Department of Obstetrics and Gynecology, Faculty of Medicine, October 6th University & Air-Force Specialized Hospital - Cairo, Egypt; 2 Obstetrics & Gynecology Department, Al-Galaa Teaching Hospital & Air-Force Specialized Hospital, Cairo, Egypt; 3 Obstetrics & Gynecology Department, Al-Azhar University & Air-Force Specialized Hospital, Cairo, Egypt

The Editorial Board has retracted this article. After publication concerns were raised about the data reported for this study. The authors provided the raw data which was examined by two independent experts who concluded that the data contain serious anomalies. The Editorial Board therefore no longer has confidence in the results presented.

